# Protein Degradation and the Pathologic Basis of Phenylketonuria and Hereditary Tyrosinemia

**DOI:** 10.3390/ijms21144996

**Published:** 2020-07-15

**Authors:** Neha Sarodaya, Bharathi Suresh, Kye-Seong Kim, Suresh Ramakrishna

**Affiliations:** 1 Graduate School of Biomedical Science and Engineering, Hanyang University, Seoul 04763, Korea; neyha19@gmail.com (N.S.); bharathi.suri@gmail.com (B.S.); 2 College of Medicine, Hanyang University, Seoul 04763, Korea

**Keywords:** deubiquitination, inhibitors, protein quality control, proteolysis, protein stabilization

## Abstract

A delicate intracellular balance among protein synthesis, folding, and degradation is essential to maintaining protein homeostasis or proteostasis, and it is challenged by genetic and environmental factors. Molecular chaperones and the ubiquitin proteasome system (UPS) play a vital role in proteostasis for normal cellular function. As part of protein quality control, molecular chaperones recognize misfolded proteins and assist in their refolding. Proteins that are beyond repair or refolding undergo degradation, which is largely mediated by the UPS. The importance of protein quality control is becoming ever clearer, but it can also be a disease-causing mechanism. Diseases such as phenylketonuria (PKU) and hereditary tyrosinemia-I (HT1) are caused due to mutations in *PAH* and *FAH* gene, resulting in reduced protein stability, misfolding, accelerated degradation, and deficiency in functional proteins. Misfolded or partially unfolded proteins do not necessarily lose their functional activity completely. Thus, partially functional proteins can be rescued from degradation by molecular chaperones and deubiquitinating enzymes (DUBs). Deubiquitination is an important mechanism of the UPS that can reverse the degradation of a substrate protein by covalently removing its attached ubiquitin molecule. In this review, we discuss the importance of molecular chaperones and DUBs in reducing the severity of PKU and HT1 by stabilizing and rescuing mutant proteins.

## 1. Introduction: Overview of Phenylketonuria and Hereditary Tyrosinemia

Phenylalanine hydroxylase (PAH) and fumarylacetoacetate hydroxylase (FAH) are two highly regulated liver enzymes that catalyze the rate-limiting step in phenylalanine and tyrosine metabolism [1,2]. Mammalian PAH (phenylalanine 4-monooxygenase, E.C. 1.14.16.1) catalyzes the stereospecific hydroxylation of L-phenylalanine into L-tyrosine using tetrahydrobiopterin (BH4), non-heme iron, and dioxygen as co-substrates in the cytosol of the liver and kidney [3]. PAH facilitates oxidation of excess L-phenylalanine into carbon dioxide and water, and is the major enzyme degrading 75% of L-phenylalanine from the diet [2]. PAH assembles as a homotetrameric protein, each subunit composed of N-terminal regulatory domain for allosteric activation by Phe, a central catalytic domain, and C-terminal helix responsible for tetramer formation [4,5].

Likewise, FAH is the last enzyme in the tyrosine catabolism pathway, and it catalyzes the hydrolysis of fumarylacetoacetate into fumarate and acetoacetate as the final step in phenylalanine and tyrosine degradation. FAH is a cytosolic dimer that consists of two *α*–*β* domains; 300 residues of the C-terminal domain form the active site that binds to Ca^2+^ and participates in intermolecular interactions at the dimer interface; 120 residues of the N-terminal domain play the regulatory role [6,7]. The FAH dimer is solely considered to be catalytically active [7]. The human *FAH* gene occupies chromosome 15q23–q25, spans 30–35 kb, and contains 14 exons [8], whereas the *PAH* gene is located on chromosome 12q at position 23.2, spans 90 kb, and contains 13 exons [9].

In 1932, Grace Medes discovered 4-hydroxyphenylpyruvate in the urine of a 49-year-old man and described it as “tyrosinosis” [10]. In the 1960s, the condition was referred to as hereditary tyrosinemia type-I (HT1), and it was later understood to result from FAH deficiency [11,12,13,14]. Deficiency of this enzyme leads to the accumulation of upstream metabolites such as fumarylacetoacetate (FAA) and maleylacetoacetate, which are subsequently converted into succinylacetone. FAA and succinylacetone are both genotoxic and carcinogenic [15]. Similarly, in 1934, Dr. Asbjørn Følling recognized elevated levels of phenylketonuric acid in the urine of two mentally retarded siblings and named the condition “phenylpyruvic oligophrenia” or phenylketonuria (PKU) [3]. Elevated levels of blood phenylalanine and its metabolites, such as keto acid and phenylpyruvate, along with reduced blood tyrosine levels, are the characteristics of PKU and its milder variant hyperphenylalaninemia (HPA). PKU is classified as classical PKU (plasma Phe levels > 1200 μM), mild or atypical or variant PKU (600–1200 μM), and non-PKU mild HPA (120–600 μM) [16,17]. PKU is associated with mental retardation, epilepsy, brain damage, and neurological and behavioral problems due to the accumulation of phenylalanine byproducts. Tyrosine is the precursor for multiple molecules; therefore, tyrosine deficiency leads to deficiency of catecholamine neurotransmitters, melanin, and L-thyroxine [3,18].

HT1 pathogenicity is largely unknown; however, missense mutations in the *FAH* gene may influence catalytic activity, protein stability, and/or protein homeostasis and monomer-dimer equilibrium [7]. Despite being studied extensively since years, the pathophysiology of PKU is not fully elucidated. Mutation-driven PAH protein instability, misfolding, and aggregation are the hallmark associated with the disease resulting in subsequent protein turnover [19,20,21]. The regulation of L-Phe by PAH is a complex mechanism associated with transition between oligomeric state, changes in conformation, phosphorylation and substrate activation, and cofactor inhibition [4,5]. The newly discovered crystal structure supports the notion that PAH exists in two native states: resting state-PAH (RS-PAH) and activated-PAH (A-PAH). The RS-PAH and A-PAH was determined by X-ray crystallography and small-angle X-ray scattering respectively [4]. The RS-PAH has low affinity for Phe and helps maintain the basal level of Phe essentially available for cellular functions. Also, BH4 is complexed with RS-PAH, thus acting as a negative regulator for L-Phe activation [4,22]. BH4 serves as a pharmacological chaperone stabilizing PAH and increasing the steady state level of enzyme [20]. As the concentration of Phe increases, excess Phe acts as an activator and binds to A-PAH allosterically, shifting the equilibrium from RS-PAH to A-PAH. Binding of Phe induces large conformational change and dimerization of regulatory domain of the enzyme, thus exposing the active site for the conversion of Phe to Tyr [4,5,22]. BH4 and Phe binding drives the newly synthesized, partially folded, PAH into equilibrium of native structure [22]. PKU disease-associated alleles affect several different operations (like allosteric activation by Phe, stabilization by BH4) that join forces for efficient degradation of excess Phe. Therefore, it is important to maintain the PAH structure equilibrium which is hampered due to disease-associated mutations.

The crystal structure of the PAH tetramer providing information about PAH allostery and BH4 associated stability was recently discovered [22,23]. The allosterically activated form of PAH is majorly responsible for the conversion of phenylalanine to tyrosine; however, stability calculations are not possible for this form as its high resolution structure is not yet available. Nonetheless, certain experimental reports suggested increased aggregation, high instability, and accelerated degradation of the PAH mutant expressed in Enu^1/1^ and Enu^1/2^ heteroallelic mouse model, primary hepatocytes and COS-7 cells [24,25,26]. The mutant PAH proteins (e.g., p.V106A) expressed in Enu^1/1^ mouse model, are also known to be highly ubiquitinated in vitro and in vivo, targeting it for proteasome-mediated degradation and selective autophagy [24].

To combat the pathogenic accumulation of defective proteins, the cells are equipped with the protein quality control (PQC) system, mainly including molecular chaperones and the ubiquitin proteasomal system (UPS). The supplementation with cofactor BH4, also acting as a pharmacological chaperone, stabilizes the PAH tetramer structure, providing a rationale for the BH4-responsive *PAH*-variants [20]. When *PAH* variants are co-expressed with GroEL/ES bacterial chaperone in *Escherichia coli*, decrease in dimer portions, increase in tetramer formation, and increase in residual activity were observed. These results suggest that co-expression with GroEL/ES bacterial chaperone might affect the PAH folding in *Escherichia coli* [19]. These results indicate that molecular chaperones have the potential to prevent protein misfolding and help to stabilize a range of mutant proteins. The proteins that cannot be stabilized by chaperones undergo degradation to avoid its interaction with other native and non-native proteins [27]. UPS is the major cellular degradation pathway, responsible for degrading more than 80% of intracellular proteins [28]. The proteins have to be tagged with ubiquitin moiety, in order to be degraded by the UPS. The PAH and FAH protein is reported to be ubiquitinated [7,24,28,29,30] and the variants are prone to aggregation and/or degradation [22]. However, the *PAH* and *FAH* variants exert some amount of residual activity depending upon the severity of mutation. Therefore, certain *PAH* and *FAH* mutants with folding defects are still functional, but they nonetheless suffer rapid degradation [21,31,32,33]. The degradative system therefore needs a way to differentiate between lethal defects and negligible defects. In this review, we discuss different strategies for stabilizing and increasing the concentrations of those functional mutant proteins, that display instability and folding defects and which are conjugated with ubiquitin molecule for degradation. We propose recruiting members of the ubiquitin proteasomal system (UPS) and protein quality control (PQC) chaperones into therapeutic endeavors to rescue functional misfolded proteins from accelerated degradation.

## 2. Etiology

More than 1000 mutations result in PKU [16], and more than 100 mutations result in HT1, and most of them are missense mutations [7]. In the *PAH* gene, 60.5% of mutations are missense mutations, and in the *FAH* gene, 45% of mutations are missense mutations [8,9]. The severity of a mutation depends on its effect on the resulting enzyme’s conformation and function. In other words, the genotypic effect on the clinical phenotype is variable [3,33]. Most of the mutations causing PKU and HT1 result in PAH and FAH instability, leading to misfolding and loss of function [20,22,33,34,35].

Because both enzymes are biallelic, it is possible to have many disease-causing mutations, resulting in a compound heterozygote [36,37]. Mutations in the *PAH* and *FAH* genes are known to decrease catalytic activity and reduce the kinetic stability of the enzymes, inducing accelerated degradation [7,38].

## 3. Epidemiology

PKU and HT1 are autosomal recessive traits that affect 1 in 2500 to 100,000 births [9,39] and 1 in 100,000 births [40], respectively. PKU has high prevalence in the United Kingdom, Turkey, and Ireland and is rare in Thailand, whereas HT1 is present worldwide except for Central America and Oceania. The most common mutation in PKU is p.Arg408Trp, which is frequently found in Russia and East European countries such as Hungary, the Czech Republic, Slovakia, and Croatia [41,42] and Baltic countries such as Estonia, Lithuania, and Latvia [42]. Mutations such as p.Arg241Cys, p.Arg243Gln, and Ex6-96A>G are frequent in the Chinese population [43], and p.Pro281Leu is common in Iranians [44]. Other common variants include p.Arg261Gln, p.Tyr414Cys, and p.Ser349Pro [42]. More than 40 mutations have been found in the *FAH* gene, and some of the most common are D233V in Turks, W262X in Finns [45], p.Gly64His in Asians, p.1 Met>Val in Saudi Arabians [8], and c.974C>T in Europeans and Caucasians [46].

## 4. Protein Quality Control

Eukaryotic cells possess a robust complement of proteins to monitor and maintain a healthy proteome for their survival. Proteome integrity is maintained under the scrutiny of the PQC system [47]. The maturation from a nascent polypeptide to a functional protein is crucial to its function and involves a multistep process, including proper post-translational modification. The folding process for some proteins starts during their synthesis itself, which is called co-translational folding, whereas other proteins fold in the cytoplasm or the endoplasmic reticulum (ER) and mitochondria after synthesis [48]. The fundamentals of protein folding are also governed by the cellular environment and its over-crowding [49]. The hydrophobic patches in a polypeptide are buried in the native state. Exposure leads to the formation of intermediates that can interact inappropriately with other molecules. Thus, several studies suggest that protein folding is initiated by composing a folding nucleus within a primary structure around which the remaining polypeptide folds. The most important requirement for a correct folding pattern is the interaction between the hydrophobic and polar residues during nucleation, which encourages the structure to be packed correctly [50].

The protein-folding mechanism is much more complex for larger proteins than for smaller ones. Evidence from various studies indicates that, during protein folding, some proteins attain the native structure, whereas others cannot, for reasons such as a non-native interaction that leads to intermediates or a transiently folded protein state. Therefore, large proteins are assembled from diverse segments or domains that are folded simultaneously and independently, ensuring the proper folding of each segment, so that they can correctly interact with one another to form a highly stable and compact, native, three-dimensional protein structure. In other words, for large protein complexes such as proteasomes and ribosomes, the folding pathway involves a two state mechanism [51,52].

**Figure 1:**
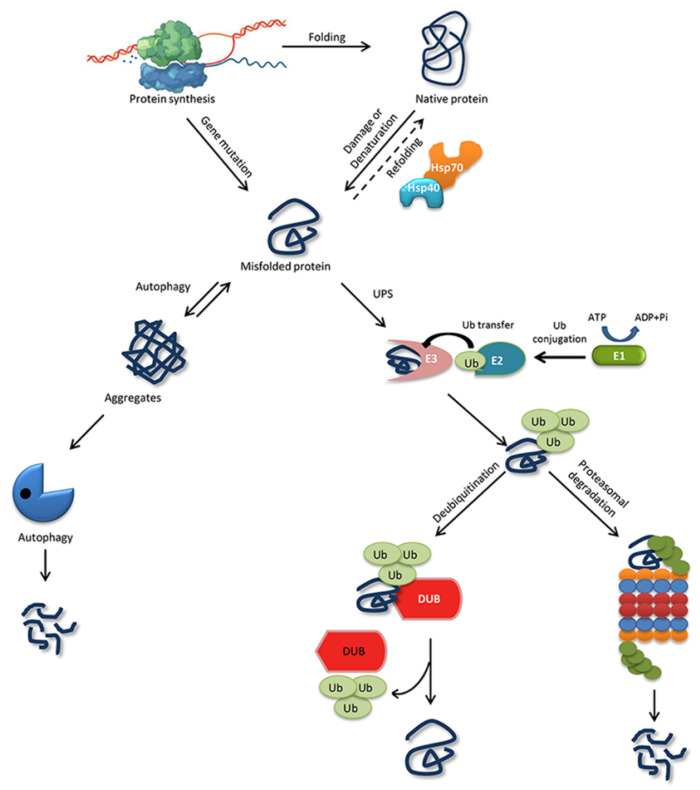
Protein folding, misfolding, and degradation. Protein folding starts during the ribosomal translation process and attain the native conformation to execute cellular processes. The native folded proteins are often misfolded due to mutations and other environmental factors. Molecular chaperones catalyze the folding/refolding events, disaggregation of the protein aggregates, and targeting the protein for degradation. Aggregates are typically degraded by autophagy, whereas the ubiquitin proteasome system (UPS) degrades the destabilized/misfolded proteins by covalent attachment of a ubiquitin molecule assisted by E1-E2-E3 enzymes. The ubiquitinated proteins are recognized by the 26S proteasome and are degraded. However, the ubiquitin moiety is cleaved off by Deubiquitinating enzymes (DUBs) and the protein can be rescued from the degradation cycle.

The ability of a protein to fold correctly de novo, though thermodynamically favorable, is often hampered by transcriptional or translational errors, destabilizing mutations, or stress conditions such as heat, oxygen radicals, aging, or environmental threats, giving rise to misfolded proteins and off-pathway aggregates [53,54,55]. The misfolded proteins can exhibit either loss of function, characterized by protein dysfunction and a propensity for degradation, or gain of function, characterized by protein aggregates that cause the misfolding of other proteins through inappropriate interactions [50,56]. Cells are rescued from the dangers of misfolded proteins by the PQC system, which keeps proteins under the constant surveillance of molecular chaperones and induces the rapid degradation of misfolded proteins through the UPS or autophagy-driven lysosomal proteolysis [57,58] (Figure 1). PQC relies on three parallel strategies whereby misfolded proteins are refolded, degraded, or delivered to a quality-control compartment capable of sequestering them, such as the juxta nuclear quality control, insoluble protein deposit, aggresome, or ER-associated degradation (ERAD) vesicles [48].

### 4.1. Molecular Chaperones for Folding/Refolding

Molecular chaperones are present in all cell organelles and can be categorized as “folding helpers” and “holding types.” A folding helper assists in polypeptide folding during translation and partially unfolds misfolded intermediates [59]. The holding type, on the other hand, guards an unfolded or misfolded protein from aggregation and degradation, and presents it to a folding helper [60]. They transiently interact with mutant proteins to protect them from interacting with normal proteins and buy time to refold them into their native conformation [27]. Most mutated proteins show a prolonged interaction with the chaperones compared with their corresponding correctly folded protein, because the chaperones need time to fold them properly [61]. However, the severity of the mutation determines whether the chaperones refold and rescue the proteins or direct them for degradation. Therefore, the ectopic expression of chaperones increases the chance that chaperones will interact with misfolded proteins and restore their active form, as observed in previous studies [62,63]. In other words, chaperones along with other members of PQC, play a decisive role in maintaining a pool of functional mutant proteins.

Molecular chaperones also help certain newly synthesized proteins to fold efficiently and in a biologically relevant time frame. Chaperones such as HSP70/40 attach to a partially folded polypeptide during synthesis by a ribosome and stabilize it to its correct native conformation [64] (Figure 1). Most proteins fold to a native form in the cytosol and do not need any further assistance from chaperones, but certain proteins always need assistance from chaperones, such as HSP90 and the TCP-1 ring complex (TRiC) [65]. Likewise, chaperones such as HSP70 and HSP90 and some chaperonins (such as HSP60s) assist in the refolding of misfolded proteins through the ATP and co-factor binding and release cycle. If a misfolded protein cannot be completely refolded, chaperones help target it for degradation to remove the toxic conformation. Co-chaperones such as the Bcl-2-associates-athanogene domain direct the interaction between a molecular chaperone and the protein degradation system [66,67].

Several classes of molecular chaperones are distinguished by molecular weight and mode of action, such as Hsp70, Hsp90, Hsp60, and Hsp40, and bind to the hydrophobic region of a non-native protein [64,68].

#### 4.1.1. Hsp70

The 70 kDa heat shock protein (Hsp70) and its homologs heat shock cognate 70 (Hsc70) in the cytosol and BiP/GRP78 in the ER are some of the most abundant chaperones engaged in a plethora of folding and refolding processes. Hsp70 consists of two domains: an N-terminal nucleotide-binding domain (NBD) of ~40 kDa and a C-terminal substrate-binding domain (SBD) of ~30 kDa, connected by a hydrophobic linker. The Hsp70 cycle can use co-chaperones such as Hsp40 to recognize and transfer a substrate protein to Hsp70, J proteins to stimulate ATP hydrolysis in the SBD, and nuclear exchange factor proteins to change ATP to ADP in the NBD. ADP-Hsp70 holds a substrate protein in an unfolded state, until it has no exposed hydrophobic patches and spontaneous folding is achieved [60,69].

#### 4.1.2. Hsp90

Hsp90 is a homodimer, and each of its monomers consists of a highly conserved amino-terminal domain (NTD) of ~25 kDa, a middle domain of ~40 kDa, and a C-terminal dimerization domain of ~12 kDa [70,71]. Hsp90 is also an ATP-dependent chaperone present in almost all the compartments of a eukaryotic cell. Hsp90 regulates the stability and maturation of >300 proteins that are key players in many biological processes, such as immune response, telomere maintenance, cancer development, steroid signaling, and vesicular transport [72]. To recognize its enormous number of client proteins, Hsp90 interacts with more than 20 co-chaperones. Hop, also known as p60, and STI1 mediate the transfer of client proteins from Hsp70 to Hsp90, and Cdc37 binds to kinase clients that inhibit Hsp90’s ATPase activity. Co-chaperone Aha1 enhances Hsp90’s ATPase activity by binding between HSP90’s middle domain catalytic loop and the NTD nucleotide-binding site, facilitating the transition to a more stable closed transformation [72]. Unlike other chaperones, Hsp90 binds to partially folded intermediate conformations rather than to fully denatured proteins [73,74].

#### 4.1.3. Hsp60

Hsp60 or chaperonins are 800–900 kDa double ring cylindrical complexes that originate in the mitochondria, but migrate to the cytosol under cellular stress. Cytosolic Hsp60 is also called TRiC or the TCP1 complex and usually has an 8-membered ring, whereas mitochondrial Hsp60 has a 7-membered ring modulated by a lid structure made of co-chaperone Hsp10. Client proteins enter the central cavity where the apical domain of Hsp60 exposes its hydrophobic residues for substrate binding. Following substrate binding, Hsp60 subunits undergo excessive conformational changes, ATP is hydrolyzed, and the folded protein is released through the Hsp10 lid [75,76]. 

#### 4.1.4. Hsp40

Hsp40, also known as J protein, is mostly studied in its function as a co-chaperone with Hsp70. The Hsp40 family is divided into 3 types depending on the location of the J chain. In types I and II, the J-chain is located at the N-terminal, whereas in type-III, the J chain can be located anywhere in the protein sequence. The type-I protein additionally possesses two zinc finger motifs. The J chain, which is 70-amino-acid-residue long, stimulates the ATPase activity of Hsp70. Hsp40 is known to recognize and bind misfolded proteins and guide them to Hsp70 for folding. Hsp40 has been shown to play a crucial role in neurodegenerative diseases and cancers; however, its mechanism for recognizing non-proteins and modulating Hsp70 activity is poorly understood [77].

### 4.2. Ubiquitin Proteasome System

Misfolded or destabilized proteins undergo intracellular proteolysis through two main pathways: the UPS and the autophagy-lysosome pathway. More than 80% of native and misfolded intracellular proteins undergo UPS-mediated degradation [28]. The UPS functions in both the nucleus and the cytosol to recycle and degrade soluble proteins. On the other hand, autophagy functions only in the cytoplasm and generally eliminates large, insoluble aggregates and degenerated organelles that escaped from the UPS [68]. A detailed explanation of the autophagy-lysosome pathway has been provided elsewhere [78]. In this review, we focus mainly on UPS-mediated protein degradation and its association with chaperones during the regulation of PAH and FAH proteins.

Targeting a substrate protein for UPS degradation requires the covalent attachment of Ub molecule/s, which is called ubiquitination. Ubiquitination is achieved when three enzyme families work consecutively: ubiquitin-activating enzyme (E1) activates ubiquitin by ATP hydrolysis and forms a thioester link between its own cysteine residue and the C-terminal carboxyl group of Ub. Second, ubiquitin-conjugating enzyme (E2) receives Ub from E1 by a trans-thiolation reaction in which Ub binds to the cysteine residue of E2. Third, the ubiquitin-protein ligase (E3) and E2 together position the target protein substrate and attach the ubiquitin moiety to the ε-amino group in a lysine residue on the target protein. The ubiquitin-conjugated proteins are then recognized and degraded by the 26S proteasome unless the ubiquitin chains are removed by a crucial set of enzymes called deubiquitinating enzymes (DUBs) [79,80] (Figure 1).

The role of the UPS has mainly been characterized in neurodegenerative diseases [81]. In gain-of-function disorders such as Alzheimer’s, Parkinson’s, Huntington’s, and Creutzfeldt-Jakob diseases, the misfolded proteins accumulate to form aggregates and fail to undergo proteasomal degradation because of dysfunction in the UPS. In Alzheimer’s disease, for example, the amyloid precursor protein is cleaved into amyloid β (Aβ) peptides to form intraneuronal neurofibrillary tangles. The Aβ peptides inhibit UPS-mediated degradation by binding to the catalytic core of the 26S proteasome, thereby inhibiting its chymotrypsin-like activity [82]. Interestingly, the molecular chaperones Hsp70 and Hsp40 reduce the accumulation of Aβ aggregates [83]. Therefore, the UPS and molecular chaperones balance each other’s functions.

## 5. Deubiquitinating Enzymes Regulate Molecular Chaperones

Approximately 100 putative DUBs have been identified in humans. These large ubiquitin-cleaving proteases are classified into seven families: ubiquitin-specific proteases, ubiquitin C-terminal hydrolases, ovarian tumor proteases, Machado-Joseph disease domain proteases, Jab1/Mpn/Mov34 metalloenzymes, monocyte chemotactic protein-induced proteases, and zinc finger with UFM1-85 specific peptidase domain proteins, but most of their functions and substrates have not yet been characterized [84]. Some of the important functions of DUBs in the ubiquitin pathway include generating free ubiquitin monomers by processing inactive ubiquitin precursors, acting as an E3 ligase antagonist by cleaving the ubiquitin molecule from the substrate proteins, and maintaining a ubiquitin pool by recycling cleaved ubiquitin molecules. DUBs are known to be involved in physiological processes and thus are predicted to be involved in cancers [85], neurodegeneration [84], and infectious diseases [86]. Interestingly DUBs also regulate the members of another major degradation pathway in PQC called autophagy. Misfolded proteins are recognized by molecular chaperones in the HSP family, which coordinates with the UPS for protein refolding and the removal of misfolded proteins [87].

Ubiquitination and deubiquitination both play crucial roles in the dynamic regulation of different stages of the autophagic process. To induce proper autophagy, post-translational modification of its initiators is essential. It is a well-organized game of “on” and “off” between the E3 ligase and DUBs in controlling autophagy signals [87]. Numerous E3-chaperone complexes work in parallel to target misfolded proteins. For example, the E3 ligase carboxy-terminus of Hsc70 interacting protein (CHIP) tightly regulates the function of Hsp70/Hsp90 to orchestrate cellular protein folding and degradation. Ubiquitination of substrate proteins is antagonized by DUBs, allowing misfolded proteins to escape from degradation [88]. A growing body of evidence suggests crosstalk between the DUBs and the HSPs as well. For instance, proteasome-bound USP14 protein was found to interact with molecular chaperone Hsc70 to modulate autophagy in neuroblastoma cells. Striatal neuronal cells expressing mutant huntingtin protein had a defect in autophagosome maturation that was influenced by Hsc70 and proteasome free-USP14, indicating a link between the proteasome-independent function of USP14 and Hsc70 in mediating crosstalk among autophagy, ER stress signaling, and the proteasome [89]. Similarly, the DUB USP19 has two major isoforms. One isoform contains a transmembrane domain at its C-terminus and is associated with ERAD for an unfolded protein response; the other isoform contains an EEVD extension at the C-terminus that interacts with CHIP. The N-terminus of both isoforms interacts with the Hsp90 chaperone. The regulatory function of USP19 was recently confirmed in a study demonstrating that USP19 interacts directly with chaperone Hsp90 and upregulates the aggregation of poly-Q containing the proteins Ataxin-3 and Huntingtin, which causes spinocerebellar ataxia type-3 and Huntington’s disease, respectively [90]. Direct evidence indicates that chaperone Hsp90 enhances USP19 DUB activity by promoting its substrate recognition [91]. Hsp90 recruits misfolded proteins for refolding, and should the protein fail to refold, the co-chaperone CHIP ubiquitinates the misfolded protein for degradation with the help of Hsp90, or the misfolded protein is deubiquitinated by USP19, allowing it to avoid degradation and promoting aggregation [90]. This process is perfectly synchronized as a defense mechanism against proteins whose aggregation is cytotoxic to the cells. However, to enhance the rescue of functional mutant proteins, understanding the regulatory mechanism of DUBs and molecular chaperones is beneficial.

## 6. Rapid Degradation of Misfolded PAH and FAH Proteins

More than 1000 variants in the human *PAH* gene are recorded in the locus-specific database *PAH*vdb (http://www.biopku.org/home/pah.asp), and certain missense mutations in the regulatory and catalytic domains cause protein instability and folding defects of the PAH protein, resulting in its rapid degradation and loss of function [31,32,92]. Thus, PKU was generally considered to be the paradigm of misfolded metabolic diseases [93]. The destabilized mutants of PAH are precisely degraded by the cellular PQC system. Mutation-dependent destabilization and accelerated proteolytic degradation are the main pathogenic mechanisms in PKU [94]. PAH is reported to be a substrate for Ub-conjugating enzyme and is likely degraded by the UPS. Døskeland et al. demonstrated that PAH isolated from rat liver is conjugated with mono- and multi-/poly-ubiquitination at its catalytic domain [29]. More recently, in an ENU^1/2^ heteroallelic mouse model of HPA, mutant PAH was highly ubiquitinated, which corresponded with an increased rate of degradation [24]. The mutant proteins were degraded more rapidly than the wild type enzyme [62]. The wild type is reported to have a half-life of 2 days in rat liver and 7–8 h in hepatoma cells; in contrast, mutants are degraded rapidly, due to the destabilization of their protein structure [29]. Molecular chaperones such as DNAJC12/HSP70 play a role in processing mutant PAH for UPS-mediated degradation or ubiquitin-mediated autophagy [28,30].

Likewise, more than 100 mutations of the *FAH* gene cause HT1. Like PAH, most of the mutations produce FAH destabilization, causing the enzyme to be rushed to the aggregation pathway. When cells expressing the FAH protein were subjected to the proteasomal inhibitor MG132, FAH protein levels were restored. Therefore, the FAH protein undergoes proteasomal degradation [7]. FAH is also conjugated with Ub at multiple lysine residues according to the PhosphoSitePlus (www.phosphosite.org) database. However, no evidence indicates the type of ubiquitin linkage and whether it targets FAH for degradation or tags it for further cellular processes. The reduced activity and deficiency of FAH found in HT1 could result from the rapid degradation of destabilized mutant proteins [95], similar to PAH in PKU.

## 7. Residual Catalytic Activity of PAH and FAH Can Be Rescued by Deubiquitination or Molecular Chaperones

Certain cases of PKU result from genetic mutations that impede the normal folding of the wild type PAH protein, leading to reduced or no enzyme activity. Genotype-based prediction of metabolic phenotypes, including patients with homozygosity and those with functional hemizygosity, has been studied for several years [16]. Two alleles, both with severe mutations in the *PAH* gene, produce an enzyme with little or no enzyme activity, whereas the presence of two mild mutations or one severe and one mild mutation produces high residual enzyme activity, producing HPA or mild PKU (>30% activity compared with wild type PAH) [93]. Certain combinations of mutations in the genotype and their predicted residual enzyme activity have already been reported [96,97]. Some mutations characterized by high residual activity were found to be responsive to natural co-factor BH4 [98]. BH4 responsiveness has a multifactorial basis, including intragenic polymorphisms and non-genetic factors. The main molecular mechanism underlying BH4 responsiveness is its chaperone-like effect on PAH, whereby it protects PAH protein integrity and rescues it from Ub-dependent degradation [99]. 

It is increasingly apparent that molecular chaperones could help mutant PAH proteins that are partially functional serve their purpose and help to prevent the pathogenic mechanisms that underlie genetic diseases. 

Given the importance of chaperones, mutations in the chaperones themselves can be lethal. Multiple diseases are associated with mutations in the regulating chaperones. For example, a missense mutation in the equatorial domain of HSP60 causes spastic paraplegia, and mutation in tubulin-specific chaperone E causes hypoparathyroidism, mental retardation, and facial dysmorphism [100]. Similarly, in PKU an autosomal recessive mutation in DNAJC12, a PAH co-chaperone, reduced the activity of wild type PAH, leading to HPA. DNAJC12 is involved in PAH folding and interacts with the monoubiquitinated *PAH* variant, marking it for the Ub-dependent proteasomal/autophagy degradation system. Further studies are ongoing to elucidate the role of DNAJC12 in regulating PAH and PAH mutants [25,101]. Gene therapy and the ectopic expression of wild type chaperones might help to restore the partially functional mutant proteins [102,103].

Some patients with HT1 who are treated with NTBC (2-[2-nitro-4-(trifluoromethyl) benzoyl] cyclohexane-1,3-dione) also suffer from chronic hepatopathy and the development of hepatocellular carcinoma [104]. In a murine model of HT1, chaperones such as HSPB and HSPA were found to be associated with the anti-apoptotic proteins BCL-2 and BAG in the hepatocarcinogenetic process [105]. However, the role of molecular chaperones in FAH protein stability and degradation needs to be investigated.

Another system that can be targeted to rescue defective proteins is the UPS. As discussed in the previous section, the UPS is mainly driven by E1-E2-E3 enzymes that tag substrate proteins with ubiquitin molecules to mark them for degradation via the 26S proteasome, and DUBs can reverse that process. The role of DUBs in disease regulation has been imagined ever since their discovery, because they are involved in almost all cellular processes [106]. In PQC, the ubiquitin-mediated proteolytic pathway is a dynamic system responsible for regulating the fate of many proteins. In loss-of-function diseases, saving functional misfolded proteins from degradation can be a better alternative than dealing with a deficiency of proteins caused by rapid degradation. DUBs can rescue proteins from degradation by cleaving their degradative signals. Thus, DUBs act as proofreaders for mis-tagged substrate proteins and prevent them from degradation. In that way, DUBs could be used to curb protein misfolding diseases. It is unsurprising that direct evidence on this point is sparse. Most studies dealing with diseases related to protein folding problems aim to clear the misfolded proteins from the cells rapidly, and thus they target DUBs or the proteasome via specific inhibitors to prevent the pathogenesis of defective protein accumulation [107,108]. However, in diseases such as PKU and HT1, artificial manipulation of those systems could prove advantageous and pave the way for new therapeutic approaches. Nonetheless, the regulation of the proteostasis is not possible for those missense mutations which are present at the active site of the enzyme and other mutations causing truncation and splice variant. Therefore, controlling the proteostasis might be favorable only to the missense mutations that are located outside the active site.

PAH and FAH enzyme proteins are ubiquitinated and degraded by the Ub-dependent system, and therefore PAH and FAH mutants with high residual enzyme activity could be deubiquitinated by DUBs, which might suffice to create an adequate supply of functional protein. However, the mutations in certain genotypes can show dramatically different disease severities. Thus, in targeting DUBs as therapeutics for diseases with misfolded protein, it is important to understand the genotype-phenotype correlation and the allelic combination of mutations present in the genotype. A great deal of work remains to be done to improve understanding of how DUBs, molecular chaperones, and their combination can help to regulate enzyme deficiencies.

## 8. Current Treatments and Currently Ongoing Research

### 8.1. Dietary Treatment

PKU is the most common inborn error of metabolism; in it, the accumulation of L-Phe causes clinical features such as mental retardation, eczema, microcephaly, and behavioral problems. Blood Phe levels, which depend on the enzymatic deficiency, dictate the severity of the clinical phenotype [37]. It has been more than 65 years since the successful establishment of dietary restrictions to treat PKU, but that diet has to be maintained for life [109]. The PKU diet is low in Phe and is supplemented with a special medicinal formula to supply vitamins, amino acids (except Phe), and minerals. However, dietary restrictions are cumbersome economically and socially and can lead to nutritional deficiencies. Moreover, adhering to dietary restrictions is difficult for older patients, resulting in increased blood Phe levels. Large neutral amino acids (LNAAs) can be included in the diet to reduce the absorption of Phe in the brain by competing with the transporter across the blood–brain and gastrointestinal barriers. Because inadequate evidence supports the long-term outcomes of LNAA treatment, it is used only as a short-term therapy. Nevertheless, dietary treatment is the foundation of PKU management upon which novel improved therapies are being developed [109,110].

Dietary restrictions are the most common treatment for both PKU and HT1 patients. Patients with HT1 are recommended to consume a low tyr/Phe diet and Nitisinone [111].

### 8.2. Nitisinone

Nitisinone (NTBC, Orfadin^®^, Swedish Orphan Biovitrum, Stockholm, Sweden) has been an effective treatment for HT1 since 1992. It inhibits an enzyme, 4-hydroxyphenylpyruvate dioxygenase (HPPD), upstream of *FAH*. Nitisinone is a well-tolerated drug, but its drawbacks include the development of corneal lesions in rats, transient thrombocytopenia, and leucopenia [104,112]. Recently, clustered regularly interspaced short palindromic repeats (CRISPR)—CRISPR-associated (Cas) systems mediated gene correction has been demonstrated in vivo and ex vivo. In a mouse model of HT1, 1 of 250 liver cells was corrected by the hydrodynamic injection of a donor oligonucleotide and a plasmid co-expressing gRNA and Cas9. However, NTBC supplementation with the CRISPR components showed better therapeutic results in mice than either treatment alone [113]. In ex vivo experiments, hepatocyte cells were collected from individual HT1 mouse models, corrected using CRISPR/Cas9 components, and implanted back to the organism. That method of replacing the mutated genotype has high efficiency, but it still requires cycles of NTBC treatment [114].

### 8.3. Enzyme Therapy

In 1980, phenylalanine ammonia lyase (PAL) was recognized as a potential treatment for PKU, because of its ability to metabolize excess Phe into less toxic products, trans-cinnamic acid, and ammonia, regardless of genotype. Enzyme replacement therapy and enzyme substitution are two other treatment strategies adopted. In enzyme replacement therapy, a functional PAH enzyme and its co-factor BH4 are delivered by orthotopic liver transplantation. Enzyme replacement is a daunting process that is useful only for the few PKU patients who need a liver transplant. Enzyme substitution by PAL is less troublesome and does not require the co-factor, because it acts directly as a substitute for deficient PAH. When injected into humans, PAL triggers a host-immune response and is degraded by proteases. To overcome that problem, PAL is conjugated with polyethylene glycol (PEG-PAL). PEG-PAL, now called pegvaliase, is in clinical trials. In 2018, pegvaliase (trade name Palynziq^®^) was approved by the FDA in the USA. However, severe adverse events were frequently observed in the clinical trials. The safety of pegvaliase is also a concern during pregnancy [115,116,117].

### 8.4. Gene Therapy

Gene therapy is a promising technique for the treatment of PKU and has been studied by several researchers. Recently, a recombinant adeno-associated virus (rAAV) containing the *PAH* gene was delivered to a mouse model of PKU. But the rAAV vector could not permanently correct liver PAH because the vector was not integrated into the genome of the hepatocytes, leading to loss of the vector during subsequent hepatocyte regeneration. When the vector containing *PAH*- and co-factor-synthesizing genes was injected into muscle, it was able to metabolize Phe to Tyr, but low gene transfer means that this approach needs further improvement [118,119]. Inactive Cas9 (dCas9) fused with the *FokI* endonuclease (*FokI*-dCas9) has recently been used to correct the p.Arg408Trp mutation in the *PAH* gene. The frequency of the corrected allele was 21.4%, making *FokI*-dCas9 a promising strategy to treat PKU [120]. Several laboratories have achieved a certain degree of success in correcting the PAH deficiencies, however none has progressed to human trials yet.

### 8.5. Tetrahydrobiopterin

The supplementation of co-factor BH4 in some PKU patients with high residual activity reduced their Phe levels. The metabolic response to BH4 improves the stability of the misfolded PAH enzyme and increases enzyme activity by reducing proteolysis. In 2007, a synthetic form of BH4, sapropterin dihydrochloride, was approved in multiple countries as an adjuvant therapy. The treatment requires that the mutant enzyme possess some amount of residual activity, which is most commonly found in patients with milder forms of PKU. Most patients with severe forms of PKU, in which the enzyme activity is null, do not respond to sapropterin treatment. Sapropterin acts as a molecular chaperone to assist with PAH folding and stability. A reduction in blood Phe of 30% or more from baseline is considered to be a sapropterin response. Chaperone therapy is a promising new approach in the treatment of PKU and HPA [110,121].

### 8.6. Microbe Therapy

Development of bacteria-based drug has been focused over the past decade. In PKU, the exploitation of gut microbiome to facilitate the degradation of Phe from the diet has shown promising results in clinical trials. Synlogic (https://www.synlogictx.com/), a Massachusetts-based biotech, reprogrammed *Escherichia coli* Nissle (EcN)—that has been isolated from human microbiome. The treatment, dubbed SYNB1618, aim to target two pathways. One Phe degradation pathway, where gene *sltA* encoding Phe ammonia lyase was inserted into the EcN chromosome. Gene *sltA* encodes a cytosolic protein, hence *pheP* gene that encodes a Phe transporter was also engineered in EcN. The second pathway was the insertion of *Proteus mirabilis* pma, which encodes L-amino acid deaminase having higher Phe break down capability. Currently SYNB1618 is in phase II clinical trial to evaluate its potential to lower blood Phe in patients with PKU [122,123] (https://clinicaltrials.gov/ Identifier: NCT03516487).

### 8.7. mRNA Therapy

Moderna (https://www.modernatx.com/), a clinical stage biotechnology company pioneering messenger RNA (mRNA) therapeutics and vaccines, have developed mRNA-3283, that encodes the human *PAH* to restore the intracellular enzyme activity in patients with PKU at an effective dose amount encapsulated within a liposome nanoparticle; mRNA-3283 is currently in preclinical development.

### 8.8. Small Molecule THERAPY

Agios Pharmaceuticals 9 (https://www.agios.com/) has developed a small molecule therapy on the hypothesis that majority of mutations in PAH prevent it from folding into its native tetrameric conformation. The small molecule stabilizes some of these mutant enzymes and has demonstrated significant reduction in blood Phe in severe PKU pre-clinical models.

### 8.9. Red Blood Cell Therapy

Recently, the lab grown red blood cells were genetically engineered to produce the enzyme PAL. Researchers at Rubius Therapeutics (https://www.rubiustx.com/) developed RTX-134, a Red Cell Therapeutic™ (RCT) product candidate, by inserting the gene encoding PAL enzyme into the blood cells to degrade toxic levels of Phe in the bloodstream. RTX-134 entered the clinical trials; however, it was recently reported that RTX-134 failed to generate any meaningful signals of efficacy.

The current and ongoing treatment and research are summarized in the Table 1 and Table 2. No optimal treatment for PKU and HT1 has yet been developed, and thus novel strategies need to be explored. It is necessary to consider each step involved—folding, assembly, refolding, and degradation—in the cellular handling of mutant PAH and FAH proteins.

## 9. Molecular and Chemical Chaperones as Current Therapeutics for PKU and HT1

Different technologies have been exploited to discover strategies for treating the most common inborn errors of metabolism, PKU and HT1. For PKU, pharmacological chaperones are currently being studied to functionally and structurally rescue misfolded proteins. Among all the compounds discovered to date, only sapropterin dihydrochloride, a synthetic form of BH4, and dietary restriction are approved and used together for tetrahydrobiopterin-responsive PKU [42]. Side effects associated with sapropterin include rhinorrhea, pharyngolaryngeal pain, diarrhea, and lower than normal Phe levels in patients younger than 6 years [127]. Enzyme replacement therapy is another strategy for treating PKU. PEG-PAL (pegvaliase) can metabolize Phe and is approved for the treatment of patients with uncontrolled blood Phe levels. However, participants in the clinical trials had adverse events, and discontinuation of the drug is recommended during pregnancy and breastfeeding [128].

For HT1, only NTBC (Nitisinone) in conjunction with a low tyrosine and phenylalanine diet has been widely used. NTBC inhibits HPPD, an enzyme upstream of FAH, but it only partially protects against liver dysfunction [105].

### Molecular Chaperones

Currently the therapeutic application of chemical chaperones and pharmacological chaperones is being used to rehabilitate misfolded proteins and restore mutant protein activity. Chemical chaperones are small-molecular-weight compounds that act as artificial chaperones to stabilize the native conformation of proteins. Chemical chaperones such as glycerol and trimethylamine N-oxide correct temperature-sensitive protein folding abnormalities in cystic fibrosis transmembrane regulator (CFTR, ΔF508), p53, viral oncogene protein pp60src, and ubiquitin activating enzyme E1. However, chemical chaperones have low specificity, which produces undesired effects, and the concentrations needed to increase protein function are so high that they are toxic to cells. Therefore, chemical chaperones are not generally used in clinical practice. On the other hand, much work has been done on pharmacological chaperones, which work at low concentrations and have high specificity [130]. This strategy has already proved effective in restoring mutant PAH activity in the form of sapropterin [131]. Pharmacological chaperones to treat Fabry disease and Pompe disease show increased enzyme activity and decreased substrate accumulation and are already in clinical trials [130]. Similar candidate molecules to stabilize PAH and FAH should be identified by using high-throughput screening of drug libraries and studying thermal protein stability. Chaperones could be one of the best candidates to manipulate because of their diverse role in protein folding, assembly, and stability. Pharmacological chaperones alone or in combination with members of the UPS system (such as DUBs and E3 ligases) could be a promising therapeutic strategy for rescuing enzyme function.

## 10. Alternative or Synergistic Approaches for Treating Diseases Caused by Destabilizing Missense Mutations

### 10.1. Screening Specific DUBs for PAH and FAH Proteins

The protein misfolding and mis-assembly in PKU and HT1 cause rapid protein degradation, which it is important to preempt. The UPS is one major pathway for intracellular protein degradation. DUBs play a central role in ubiquitin signaling and protein homeostasis. The development of DUB inhibitors has been showcased as a promising strategy for treating cancers and other diseases by enhancing the degradation of DUB substrates. No one has reported on the role of DUBs in misfolding diseases, in which treatments need to stabilize and increase the concentration of mutated proteins. DUBs can recognize different chain linkages that are specific for particular functions, such as the K6, K11, and K48 chains for proteasomal degradation and K63 chains for lysosomal targeting, DNA repair, and NF-κB activation. Thus, DUBs are highly specific in their action [106].

Any misfolded protein is tagged with mono-/poly-ubiquitination chains that mark them for proteasomal degradation. Those degradation tags can be recognized and removed by DUBs, thus aborting the protein degradation cycle. Therefore, we hypothesize that DUBs could play a decisive role in rescuing functional but misfolded PAH and FAH proteins from degradation. Instead of DUB inhibitors, drugs to enhance the function of DUBs that specifically regulate PAH and FAH protein degradation need to be identified. Various DUBs might be suitable for interacting with PAH and FAH proteins to dissociate ubiquitin molecules and maintain the relatively small amount of functional protein needed in the correct subcellular destination to prevent disease phenotypes.

Ernst et al. proposed a highly active viral DUB derived from the protease domain of Epstein-Barr virus, the BPLF1 gene called EBV-DUB, which removed ubiquitin chains and stabilized misfolded proteins in 293T cells. EBV-DUB was less toxic to cells than proteasome inhibitors, because no ubiquitylated proteins accumulated. Because the ubiquitylated proteins did not accumulate, the pool of free Ub was also maintained in the cells. Proteasomal inhibitors reduce protein synthesis with even a short exposure, whereas EBV-DUB did not immediately block the translation, again giving it an advantage over proteasomal inhibitors [132]. Inhibiting proteasomal degradation can derange many other cellular processes. Of all the attempts to stabilize the PAH and FAH mutant proteins, this approach should be an important focus of research and surveillance. 

### 10.2. Modified PROTAC Technology to Use DUBs to Stabilize Partially Functional PAH and FAH Proteins

**Figure 2:**
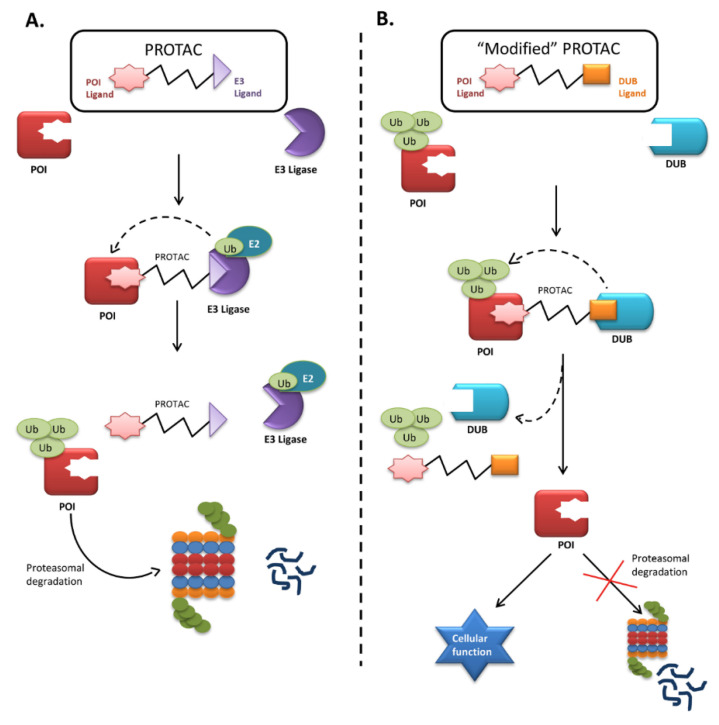
Mechanism of proteolysis-targeting chimera (PROTAC) and “Modified” PROTAC. (**A**) Proteolysis-targeting chimaera (PROTAC) are bifunctional molecules, whose one end binds to the protein of interest (POI) while the other recruits E3 ligase forming a ternary complex. The E3 ligase induces proximity-induced ubiquitination of POI by transferring the ubiquitin (Ub) molecules from E2 enzyme to the POI, thus facilitating its degradation; (**B**) Representation of a hypothetical figure where technology can be modified to rescue the functional misfolded proteins undergoing rapid degradation in inherited metabolic disorders like PKU and HT1 having partial functions, causing deficiency of available protein for cellular functions. Hence, a PROTAC can be designed whose one end binds to the misfolded POI and the other binds to a ligand that can recruit the regulatory DUB. The DUBs will cleave off the ubiquitin molecule, avoiding the protein degradation, and will help to maintain a pool of protein required for normal cellular function.

Small-molecule induced protein degradation using proteolysis-targeting chimeras (PROTACs) is an emerging technology targeting a broad range of proteins. This technology is based on event-driven pharmacology in the sense that it degrades a target protein as soon as the drug transiently binds to the target. After binding and degrading a protein, the PROTACs can again serve their function for multiple rounds of activity. PROTACs are bifunctional molecules that bind to the protein of interest with one end and to an E3 ligase with the other. The bound E3 ligase then attracts the E2 enzyme to transfer Ub to the protein of interest, targeting it for proteasomal degradation (Figure 2). Thus, this technology uses proximity-induced ubiquitination and degradation and has minimal off-target effects at a low concentration. PROTACs have already proved successful in an acute myeloid leukemia xenograft model and a disseminated lymphoma mouse model [133,134]. Therefore, they might also be used to remove null mutant PAH and FAH proteins from cells.

The recent success of small-molecule PROTACs has opened the door to a wide range of applications. This technique to induce protein degradation might also rescue proteins from degradation with some modifications. One end of the PROTAC could be designed to bind to the protein of interest, while the other, instead of binding to the E3 ligase, could be designed to bind to specific DUBs to regulate the target protein. Binding to the PROTAC would thus bring a ubiquitin-tagged misfolded protein and its DUB into close proximity, allowing the DUB to recognize and remove the ubiquitin tag and aborting the degradation (Figure 2). DUBs are usually high-molecular-weight proteins, which might give the modified PROTAC less permeability. Therefore, small molecular ligands that can recruit a specific DUB could be used instead. Due to their high specificity and low toxicity, PROTACs can be modified into selective rescuers of misfolded proteins that would otherwise be rapidly degraded. Therefore, efforts should focus on identifying a specific DUB candidate or small molecule ligand to attract DUBs that regulate PAH and FAH proteins and designing a PROTAC suitable for freezing the degradation process.

### 10.3. Inhibitor of Ubiquitin

Many studies have reported the development of novel inhibitors to target druggable enzymes of the UPS. However, the most important molecule, ubiquitin, has not yet been targeted. Recently, Nguyen et al. discovered a compound, Congo red, with the ability to bind to the recognition site and disrupt the binding activity of ubiquitin, thus making it unavailable for ubiquitination. Congo red inhibits the conjugation of K48- and K63-linked polyubiquitination. Inhibiting the ubiquitin itself inhibits all ubiquitin-mediated proteasomal/autophagic degradation, and it can therefore be used to enrich the population of all functional mutant proteins in a cell [135]. The ubiquitin inhibitor, Congo red, by itself or in combination with another strategy, could be important in supporting the survival of functional but misfolded PAH and FAH proteins.

### 10.4. E1, E2, E3 Inhibitors

Most UPS-associated inhibitors block a specific upstream component, such as E1, E2, E3, or DUBs. Those inhibitors have proved to be successful anticancer drugs, some of which are currently in clinical trials. However, E3, the last enzyme in the ubiquitin cascade, which is responsible for transferring Ub to the substrate protein, is more specific than E1 and E2, and thus an excellent target. A nutlin inhibitor of MDM2-p53, an LCL161 inhibitor of XIAP, and an ALRN6924 inhibitor of MDM2-p53 are some of the E3 enzyme inhibitors currently in clinical trials [136]. Blocking the E3 ligase with specific inhibitors will block the ubiquitination and eventual degradation of the substrate protein, which is exactly what is needed to treat enzyme deficiencies caused by the rapid degradation of functional but misfolded proteins.

## 11. Conclusions

Although a substantial body of data has been collected on the cellular mechanisms of PAH and FAH protein stability and folding, there remains a major gap in our understanding of how critical components of the PQC system recognize folding defects. The present crystal structure of PAH tetramer increments our understanding of the equilibrium between the alternate native PAH structures (RS-PAH and A-PAH), allosteric binding, and activation by Phe, and the negative regulation and stabilization by BH4 expands our knowledge of the repertoire of disease-associated PAH. There are several excellent reviews describing the structural dynamics and function of the PAH protein. However, our review is the first attempt describing the role of ubiquitination and/or molecular chaperones in PAH protein turnover. Current efforts to identify successful therapeutic strategies to treat PKU and HT1 still depend on dietary restrictions. Enzyme replacement therapy, gene correction by CRISPR/Cas9, and mRNA therapy are some recently developed breakthrough therapies associated with substantial limitations or problems. PKU and HT1 are complex diseases, with many cellular entities interacting with the substrate proteins (PAH and FAH) and their corresponding mutants, and thus it is laborious to study each of them. However, DUBs and molecular chaperones play a significant role in the triage of misfolded ubiquitinated proteins. Molecular chaperones are already being tested as therapeutic targets, but DUBs are also an attractive target. Even though research supporting the role of DUBs in rescuing the functional mutant proteins is in its infancy, future studies should be directed toward identifying and modulating protein-specific DUBs. As PKU and HT-1 are complex trait diseases, the genotype phenotype correlation plays a significant role in the development of patient-tailored prognostic and therapeutic strategies. Molecular chaperones and DUBs, alone or in combination with other therapies already established for PKU and HT1, have a bright future in treating these proteopathies. Efficient technologies and multidisciplinary methodologies are needed to explore the strong link between molecular chaperones and DUBs and how it can be used to enrich functional mutant proteins and provide new insights into proteopathy medicine.

## Figures and Tables

**Table 1:** Therapeutic regimes in phenylketonuria (PKU) and hereditary tyrosinemia-I (HT1).

Disorder	Treatment	Advantages	Disadvantages	Stage of Development	Reference
PKU	Dietary Treatment	Mainstay of treatment for PKU Successful in curtailing intellectual disability and achieving near normal IQ	Compliance due to unpalatability Nutritional deficiency Expensive	Clinical application	[109,124]
	LNAA	Reduce cerebral Phe concentrations Effective in maintaining acceptable plasma Phe concentrations	Compliance to restricted diet Unsatisfactory organoleptic properties Suitable only for adults	FDA approved	
	Enzyme Therapy (Palynziq)	PAL is a monomer and requires no cofactors	Suitable only for adults May cause anaphylaxis Injection site reactions	FDA approved	[125,126]
	Gene Therapy	Supplement or replace defective *PAH* gene	Immune rejection of adenovirus-transduced hepatocytes High dose needed Gender dependent effect	Research	[118,119,120]
	BH4 or sapropterin dihydrochloride (Kuvan)	Improves stability of enzyme Increase enzyme activity	Effective for BH4 responsive PKU	FDA approved	[42,127,128]
HT1	Nitisinone (NTBC)	Well tolerant drug Posses long half life	Development of corneal lesions in rats, transient thrombocytopenia, and leucopenia	FDA approved	[104,112,129]
	Gene Therapy	Supplement or replace defective *FAH* gene	Requires NTBC treatment	Research	[113,114]

**Table 2:** Emerging trends in PKU management.

Disorder	Treatment	Biotech/Pharmaceutical Company	Stage of Development
PKU	Microbe therapy (SYNB1618)	Synlogic (https://www.synlogictx.com/)	Phase II clinical trail
	mRNA therapy (mRNA-3283)	Moderna (https://www.modernatx.com/)	Preclinical development
	Small molecule therapy	Agios Pharmaceuticals (https://www.agios.com/), Camp4 (https://www.camp4tx.com/)	Preclinical development
	Red blood cell therapy (RTX-134)	Rubius Therapeutics (https://www.rubiustx.com/)	Discontinued in Phase 1b trial

## Abbreviations

PKU	Phenylketonuria
HT1	Hereditary tyrosinemia type 1
PAH	Phenylalanine hydroxylase
FAH	Fumarylacetoacetate hydroxylase
BH4	Tetrahydrobiopterin
PQC	Protein quality control
UPS	Ubiquitin proteasome system
HSP	Heat shock protein
DUBs	Deubiquitinating enzymes
ERAD	Endoplasmic reticulum-associated degradation
PROCTAC	Proteolysis-targeting chimaeras
NTBC	2-[2-nitro-4-(trifluoromethyl)benzoyl]cyclohexane-1,3-dione
A-PAH	Activated PAH
RS-PAH	Resting state PAH
